# Expression of BMP and Actin Membrane Bound Inhibitor Is Increased during Terminal Differentiation of MSCs

**DOI:** 10.1155/2016/2685147

**Published:** 2016-10-23

**Authors:** Christian G. Pfeifer, Alexandra Karl, Arne Berner, Johannes Zellner, Paul Schmitz, Markus Loibl, Matthias Koch, Peter Angele, Michael Nerlich, Michael B. Mueller

**Affiliations:** Department of Trauma and Orthopaedic Surgery, University Clinic Regensburg, Regensburg, Germany

## Abstract

Chondrogenic differentiating mesenchymal stem cells (MSCs) are mimicking embryonal endochondral ossification and become hypertrophic. BMP (bone morphogenetic protein) and Activin Membrane Bound Inhibitor (BAMBI) is a pseudoreceptor that regulates the activity of transforming growth factor-*β* (TGF-*β*) and BMP signalling during chondrogenesis. Both TGF-*β* and BMP signalling are regulators of chondrogenic cell differentiation. Human bone marrow derived MSCs were chondrogenically predifferentiated in aggregate culture for 14 days. Thereafter, one group was subjected to hypertrophy enhancing media conditions while controls were kept in chondrogenic medium until day 28. Histological evaluation, gene expression by PCR, and Western blot analysis were carried out at days 1, 3, 7, 14, 17, 21, and 28. A subset of cultures was treated with the BMP inhibitor Noggin to test for BMP dependent expression of BAMBI. Hypertrophic differentiated pellets showed larger cells with increased collagen 10 and alkaline phosphatase staining. There was significantly increased expression of BAMBI on gene expression and protein level in hypertrophic cultures compared to the chondrogenic control and increased BMP4 gene expression. Immunohistochemistry showed intense staining of BAMBI in hypertrophic cells. BAMBI expression was dose-dependently downregulated by Noggin. The pseudoreceptor BAMBI is upregulated upon enhancement of hypertrophy in MSC chondrogenic differentiation by a BMP dependent mechanism.

## 1. Introduction

The healing capacity of cartilage is very limited and therefore various tissue engineering approaches have been investigated to create pheno- and genotypically stable articular cartilage. Mesenchymal stem cells (MSCs) are promising candidates for the use of cell based tissue engineering applications. The chondrogenic potential of MSCs has been shown in different matrix-free and matrix based cell culture systems [1–5]. However, chondrogenic differentiating MSCs express markers like collagen type X, alkaline phosphatase (ALP), and MMP-13 [6–11], indicating hypertrophic conversion. This behaviour of chondrogenic differentiating MSCs mirrors the developmental pathway of growth plate chondrocytes during endochondral ossification. Additional characteristics of terminal differentiation like vascular invasion and matrix calcification have also been observed after in vivo transplantation of human chondrogenic MSC pellet cultures into mice [12, 13]. This hypertrophic conversion of chondrogenic differentiating MSCs raises concerns for a tissue engineering application of MSCs in articular cartilage repair. It is important to better understand the mechanisms that regulate late differentiation steps in chondrogenic differentiating MSCs to find ways to inhibit hypertrophy. The similarity of MSC chondrogenesis and embryonic endochondral ossification indicates that similar mechanisms are involved in both biological processes [14]. The different steps of endochondral bone development are regulated by a number of signalling molecules including bone morphogenetic proteins (BMPs), transforming growth factor-*β* (TGF-*β*), fibroblast growth factors (FGFs), parathyroid hormone-related peptide (PTHrP), Indian hedgehog (Ihh), and Wnts (Wingless-related integration sites) [15, 16].

The TGF-*β* superfamily consists of signalling molecules including TGF-*β*, BMPs, activins, inhibins, and growth and differentiation factors (GDFs). These growth factors have been implicated in the regulation of various processes during embryonic development including cell growth and differentiation, pattern formation, and tissue specification [17, 18].

BMPs form a subgroup within the TGF-*β* superfamily. BMPs are dimeric proteins and more than 20 BMP related proteins have been characterized. In the main signalling pathway, BMPs bind to a heterodimeric receptor complex composed of type I and type II serine/threonine kinase receptors [19, 20]. Upon ligand binding, type II receptor phosphorylates type I receptor.

The pseudoreceptor BAMBI (BMP and activin membrane bound inhibitor) is a transmembrane protein with structural similarity to type I receptors of the TGF-*β* superfamily but has a shorter intracellular domain. Lack of this intracellular serine/threonine kinase domain precludes enzymatic activity [21, 22]. BAMBI inhibits TGF-*β* and BMP signalling by blocking the interaction between type I and type II receptors [21]. Further on BAMBI is tightly coexpressed with BMP4 during embryonic development and may act as a negative feedback regulator of BMP signalling [21, 22]. BMP4 induction has been shown to be an important factor in the enhancement of hypertrophy in MSC chondrogenesis [23]. Finally, BAMBI mediates a considerable degree of crosstalk between the BMP signalling pathway and TGF-*β* signalling pathways.

Interestingly Chen et al. [24] found no developmental defects in mice lacking alleles for BAMBI. These transgenic mice were viable and fertile and did not show discernible developmental defects [24]. In contrast Guillot et al. [25] found swollen cells in myocardial and glomerular capillaries in BAMBI deficient mice. Most importantly in respect of limb development and the role of BAMBI in terminal differentiation of growth plate chondrocytes, Montero et al. [26] described the role of BAMBI during limb morphogenesis. Upon repression of BAMBI expression by activin, an increase in SMAD 1, 5, and 8 could be observed, which was followed by formation of ectopic cartilage.

Therefore this study was set up in order to investigate the expression pattern and possible downstream effects of BAMBI in late stage chondrogenesis. We therefore used an established hypertrophy model [14] in human MSCs using triiodothyronine as well as a refined hypertrophy model using BMP4 [23] for hypertrophic conversion of chondrogenically differentiated MSCs.

## 2. Material and Methods

### 2.1. Isolation of MSCs

Upon approval by the local ethics committee and written consent by the donors human MSCs were isolated from iliac crest bone marrow aspirates of seven male patients, aged 21 to 42 years, undergoing surgery that required autologous bone grafting from the iliac crest. MSCs were isolated by Ficoll (Biochrom) gradient centrifugation followed by polystyrene adhesion. Cells were expanded in Dulbecco's Modified Eagle's Medium (DMEM) low glucose (Invitrogen) with 10% fetal calf serum (PAN Biotech GmbH) and 1% penicillin/streptomycin (Invitrogen) at 37°C with 5% CO_2_. Growth medium was changed twice a week and cells were trypsinized at 80% confluence and frozen for later use in liquid nitrogen. After thawing and monolayer expansion, cells were used for the experiments at passage 1.

### 2.2. Chondrogenic Differentiation and Enhancement of Hypertrophy

For differentiation experiments MSCs were trypsinized and seeded in V-bottomed 96-well polypropylene plates at 200,000 cells per well in order to form 3D aggregates. Aggregates were assembled by centrifugation at 250 ×g for 5 min and chondrogenically differentiated in standard chondrogenic medium (chon) containing DMEM with high glucose (Invitrogen), 1% ITS (Sigma Aldrich), 50 *μ*g/mL ascorbate-2-phosphate (Sigma Aldrich), 40 *μ*g/mL L-proline (Sigma Aldrich), 100 nM dexamethasone (Sigma Aldrich), 1 mM sodium pyruvate (Invitrogen), and 10 ng/mL TGF-*β*1 (R&D Systems).

**Figure 1:**
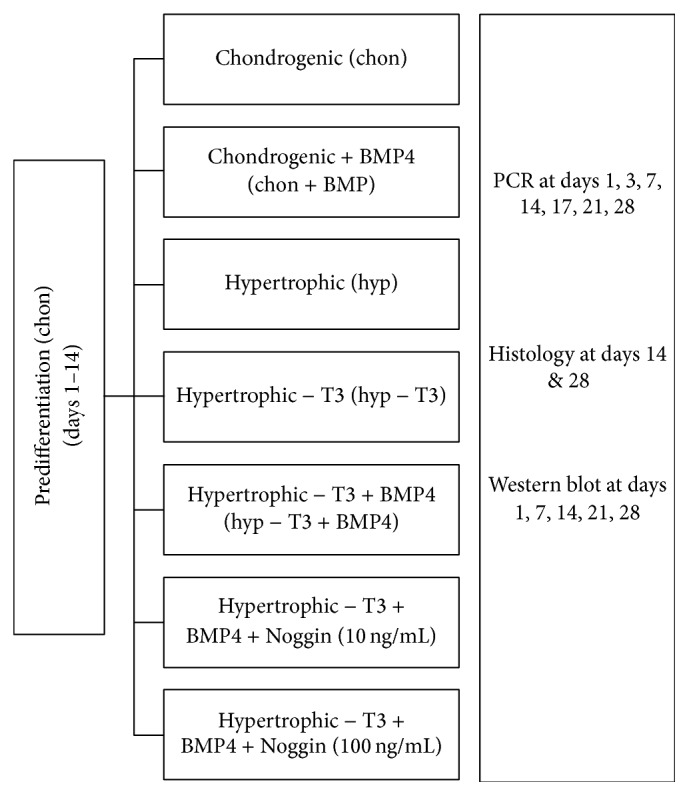
Schematic of groups and treatments: left bar shows chondrogenic predifferentiation in TGF-*β* containing medium from days 1–14. Midline bars show treatment of groups after chondrogenic predifferentiation from day 14 to day 28. Right column shows outcome measurements in regard of time points. Overall culture duration after expansion was 28 days.

Aggregates were predifferentiated for 14 days. Medium conditions were then changed and aggregates were distributed in five different groups: (1) standard chondrogenic medium (chon); (2) chondrogenic medium with BMP4 (chon + BMP); (3) standard hypertrophy enhancing medium (chon without TGF-*β* and without dexamethasone, including 1 nM triiodothyronine (T3) (Sigma Aldrich) (hyp)); (4) hypertrophy enhancing medium without T3 (hyp − T3); (5) hypertrophy enhancing medium with BMP4 instead of T3 (hyp − T3 + BMP; BMP4 was used at 100 ng/mL) (Figure 1).

In order to test for BMP4 dependent regulation of BAMBI, the BMP inhibitor Noggin was employed at 10 ng/mL and 100 ng/mL from day 1 in aggregates treated by hyp − T3 + BMP protocol (Figure 1).

Aggregates were harvested at d1, d3, d7, d14, d17, d21, and d28 for gene expression analysis.

### 2.3. Histology

Aggregates for histological analysis were harvested on d14 and d28, fixed in 4% paraformaldehyde. 10 *μ*m thick frozen sections were cut on a cryomicrotome (HM 500 OM Cryostat; Microm, Berlin, Germany). Sections were stained with 1,9-dimethylmethylene blue (DMMB) (Sigma Aldrich) for sulphated glycosaminoglycans (GAGs).

### 2.4. Histochemistry and Immunohistochemistry

For histochemical investigations 10 *μ*m thick sections were prepared as well. Histochemical ALP staining was performed with an alkaline phosphatase kit (Sigma Aldrich) with neutral red as counterstain.

For immunohistochemistry mouse anti-BAMBI (1 : 10, eBioscience), rabbit anti-BMP4 (1 : 250, Abcam), mouse anti-collagen type X (1 : 20, Quartett Immunodiagnostika und Biotechnologie GmbH), and mouse anti-collagen type II (1 : 100, Calbiochem) antibodies were used and immunohistochemistry was carried out as follows: after rinsing samples in washing buffer for 5 minutes blocking of endogenous peptidases (3% H_2_O_2_/10% methanol in PBS) was performed for 30 minutes. Then sections were incubated in blocking buffer (10% fetal bovine serum/10% goat serum in PBS) for 60 minutes at RT followed by incubation anti-BAMBI primary antibody in blocking buffer overnight at 4°C. Immunolabeling was detected by a biotinylated secondary antibody (1 : 100; Dianova), horse reddish peroxidase conjugated streptavidin (Vector Laboratories, Burlingame), and metal enhanced diaminobenzidine as a substrate (Sigma Aldrich).

### 2.5. RNA Isolation, cDNA Synthesis, and Gene Expression Analysis

For each of the 7 different independent donors 8 to 10 aggregates per condition and time point were pooled for the experiments, homogenized in 1 mL TRI Reagent (Sigma Aldrich) using the Power Gen 1000 homogenizer (Fischer Scientific), and RNA was isolated by the Trizol method. Reverse transcription was performed with Transcriptor First Strand cDNA Synthesis kit (Roche). Semiquantitative real-time PCR was performed with Brilliant SYBR Green QPCR mix (Stratagene) and the Mx3000P QPCR System (Stratagene). Gene expression was normalized to hypoxanthine guanine phosphoribosyltransferase (HPRT).

For real-time PCR the primers in Table 1 were used.

### 2.6. Western Blot Analysis

For Western blot analysis, the following antibodies were used: mouse anti-BAMBI (1 : 1000, eBioscience) and rabbit anti-*β* actin (1 : 10000, Abcam).

5 to 8 MSC pellets per time point and per condition for each patient were pooled, washed in ice cold PBS, and homogenized in 500 *μ*L 6 M urea/2% SDS solution containing a protease inhibitor cocktail (Sigma) and a phosphatase inhibitor (Sigma) using the Power Gen 1000 homogenizer (Fischer Scientific). The lysate was centrifuged for 5 minutes at 1000 ×g (4°C) and the supernatant was transferred to a new tube. The protein concentration of the supernatant was determined using the BCA Protein Assay kit (Biorad, DC Protein Assay) according to the manufacturer's instructions.

Lysates were supplemented with 4x LDS sample buffer (Invitrogen) and 10 mM dithiothreitol (DTT) and proteins were denatured for 5 minutes at 95°C. For gel electrophoresis, equal amounts of protein (10 *μ*g) were loaded and separated on a 4–12% Bis-Tris Gel (Novex by Life Technologies) at 120 V. After gel electrophoresis proteins were transferred from the gel to polyvinylidenfluoride (PVDF) membrane (Millipore). Blotting was performed for 2 hours at 100 V. After transfer, the membrane was blocked for 1 hour in 5% skim milk powder in Tris buffered saline with Tween 20 (TBST). The membrane was then incubated in primary antibody in 5% skim milk powder in TBST over night at 4°C. The next day, the membrane was washed three times for 10 minutes in TBST and afterwards incubated in HPR-coupled secondary antibody (1 : 1000, Pierce) in 5% skim milk powder in TBST at room temperature for 1 hour. The membrane was washed three times in TBST for 10 minutes. Chemoluminescence was detected with the ECL western kit (Pierce) and by using X-ray sensitive films (ECL Hyperfilm, Amersham). The films were developed in a photo developer (Curix 60, AGFA).

Western blot membranes were stripped using ReBlot Plus (Millipore) according to manufactures instructions.

### 2.7. Statistical Analysis

Gene expression was analysed by calculating the means of every relative expression normalized to the housekeeping gene HPRT. After check for normal distribution by Kolmogorov-Smirnov testing two-tailed Student's *t*-test was used. To maintain an overall *p*-level of *p* < 0.05, post hoc Bonferroni testing was carried out.

## 3. Results

### 3.1. Induction of Hypertrophy

**Figure 2:**
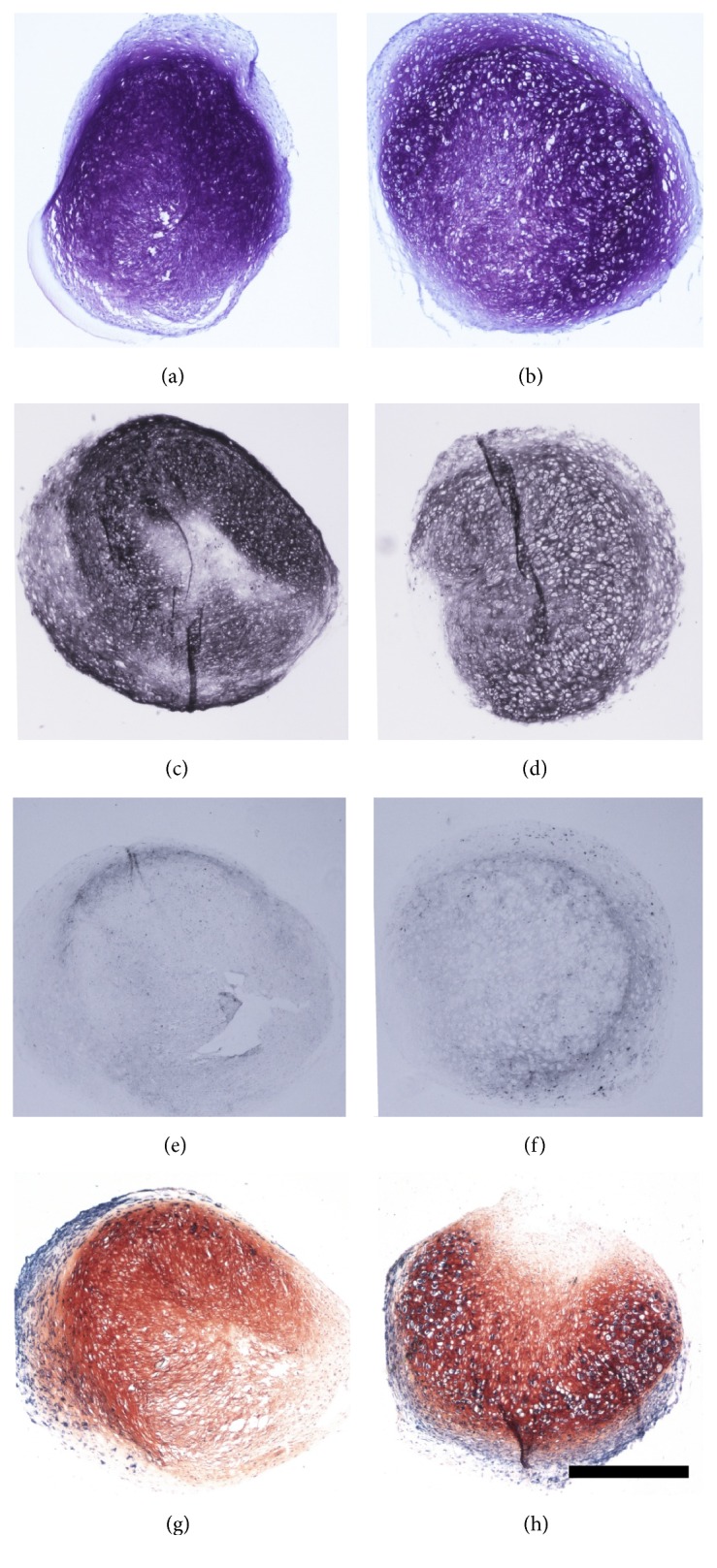
Differences in glycosaminoglycan content shown by DMMB staining of cell pellets after chondrogenic (chon) (a) and hypertrophic (hyp) conditions (b), as well as different collagen 2 production as revealed by immunohistochemistry against collagen 2 of cell pellets after chondrogenic (c) and hypertrophic conditions (d). Enhancement of hypertrophy shown by immunohistochemistry against collagen 10 between chondrogenic (e) and hypertrophic (f) conditioned cell pellets as well as by ALP staining of chondrogenic (g) and hypertrophic (h) conditioned cell pellets. Scale bar = 500 *μ*m.

Induction of hypertrophy was achieved by addition of T3 and withdrawal of dexamethasone after chondrogenic predifferentiation. The enhancement of hypertrophy was shown by an increased cell size, stronger collagen type X staining, and higher ALP activity under prohypertrophic conditions compared to standard chondrogenic conditions. Extracellular matrix analysis revealed poorer glycosaminoglycan content (Figures 2(a) and 2(b)). Immunohistochemically we observed equal to less collagen type 2 staining (Figures 2(c) and 2(d)) and analysis for hypertrophic markers showed increased collagen type 10 staining (Figures 2(e) and 2(f)) as well as strongly increased staining for the preapoptotic marker alkaline phosphatase (Figures 2(g) and 2(h)) for samples after hypertrophic conversion compared to nonconverted samples. These findings are in line with previously described characteristics of hypertrophic chondrocytes found in the growth plate [5, 14, 27], allowing using this hypertrophy model for further analysis. Both collagen type 2 and type 10 expression increases over time in both groups without significant difference between the groups (Supplementary Figure 1 in Supplementary Material available online at http://dx.doi.org/10.1155/2016/2685147). This is not concordant with the immunohistochemical results, which show higher collagen type 10 deposition in the hypertrophic group. This is a known phenomenon in this model and has also been discussed in previous papers [14]. The reason for this discrepancy is that we are dealing with mixed cell populations and not all cells respond to the switch to hypertrophy enhancing medium to the same extent. While some cells undergo hypertrophic differentiation, the TGF-*β* withdrawal may lead to dedifferentiation in other cells. This heterogeneous response can explain the missing significant difference in gene expression levels.

### 3.2. Gene Expression of BAMBI Is Increased upon Hypertrophic Conversion

**Figure 3:**
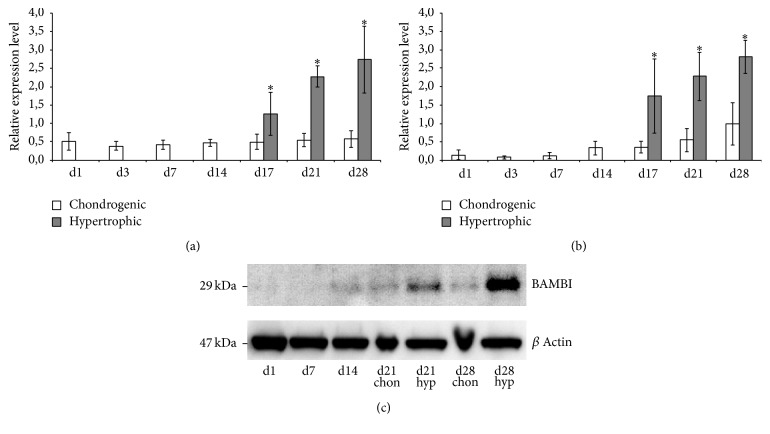
(a) Gene expression analysis of BAMBI and (b) BMP4 normalized to HPRT in MSC pellet cultures under chondrogenic and hypertrophy enhancing conditions analysed by real-time PCR. BAMBI and BMP4 are significantly upregulated under hypertrophic conditions compared to chondrogenic controls on days 17, 21, and 28. *n* = 7 different donors, ^*∗*^ *p* < 0.001. (c) Western blot analysis of BAMBI: increased amount of BAMBI protein can be detected under hypertrophic conditions on days 21 and 28 compared to chondrogenic conditions.

Real-time PCR analysis of BAMBI revealed a pronounced increase in BAMBI gene expression under hypertrophic (hyp) conditions compared to chondrogenic (chon) conditions. BAMBI gene expression is significantly upregulated at day 17, day 21, and day 28 in hypertrophic stimulated MSC pellets compared to chondrogenic pellets (Figure 3(a)). Similarly gene expression of BMP4 is also upregulated in hypertrophic conditions and reaches significant difference compared to chondrogenic conditions (Figure 3(b)). All gene expressions were normalized to the housekeeping gene HPRT.

To confirm this result on protein level we performed Western blot analysis for BAMBI on days 21 and 28. Again, in hypertrophic stimulated MSC pellets strong signals for BAMBI protein could be detected, while under chondrogenic conditions no signal was found. Remarkably, neither BAMBI gene expression nor BAMBI protein levels changed significantly under chondrogenic conditions throughout the whole time course of our experiment.

### 3.3. Immunohistochemical Staining of BAMBI Is Increased upon Hypertrophic Conversion

**Figure 4:**
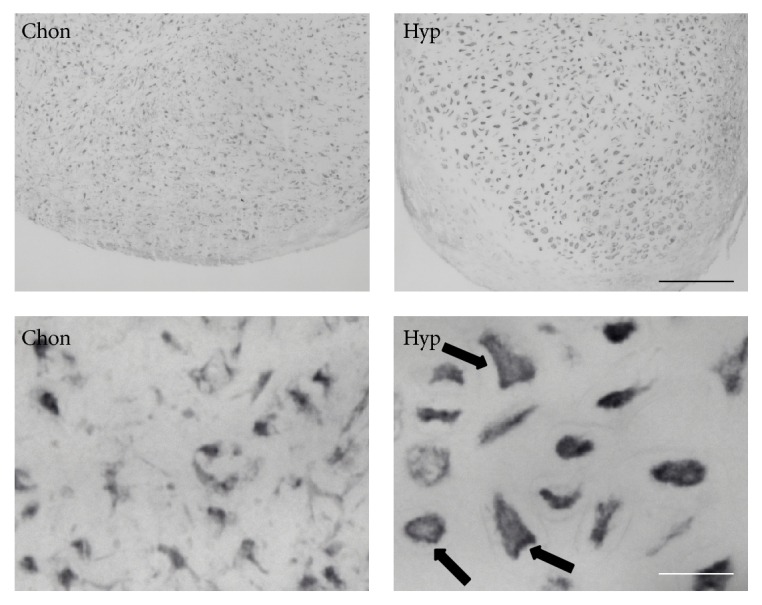
Immunohistochemistry for BAMBI. BAMBI staining is increased under hypertrophic conditions compared to chondrogenic conditions. Pronounced staining at cell membranes is detected especially under hypertrophic conditions (black arrows). Black scale bar = 200 *μ*m; white scale bar = 50 *μ*m.

To further prove the increase of BAMBI expression, immunohistochemistry for BAMBI protein was performed. Thereby we also looked for the intracellular distribution of BAMBI protein. Immunohistochemistry revealed increased staining for BAMBI in hypertrophic aggregates compared to chondrogenic aggregates on day 28 (Figure 4). In terms of intracellular distribution of BAMBI, we observed pronounced staining of the cell membranes (black arrows, Figure 4). As BAMBI is a membrane bound protein this finding was expected and confirms the presence of BAMBI protein within the cells' membranes.

### 3.4. Gene Expression of BAMBI Is Induced by BMP4

**Figure 5:**
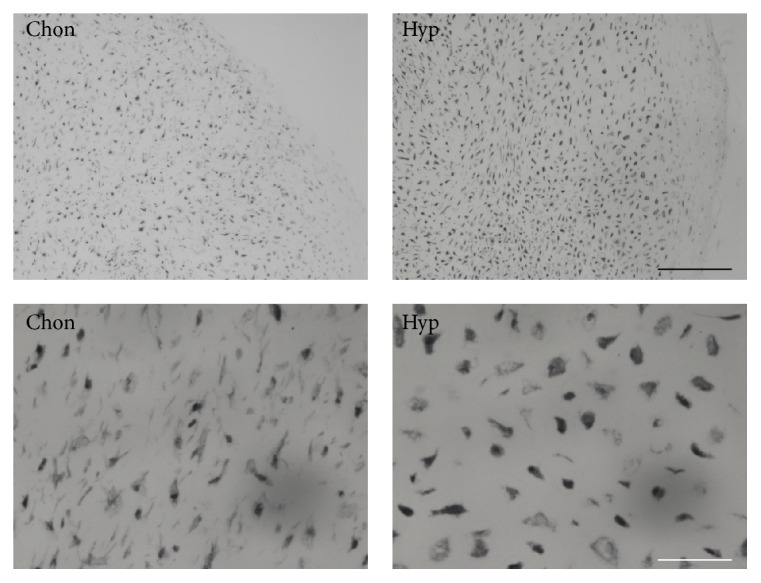
Immunohistochemical staining of BMP4 of day 28 MSC pellets. BMP4 protein staining is increased under hypertrophy (hyp) enhancing conditions as compared to chondrogenic (chon) conditions. Black scale bar = 200 *μ*m; white scale bar = 50 *μ*m.

To further investigate the role of BAMBI in the induction of hypertrophy, we analysed the expression of BAMBI after BMP4 and Noggin treatment. It was previously shown that induction of hypertrophy by T3 and withdrawal of dexamethasone and TGF-*β* is mediated by BMP4 [23]. This is also revealed by increased BMP4 protein staining in hypertrophically converted groups compared to chondrogenic controls (Figure 5). Therefore we suggested that similar expression patterns for BAMBI are achieved upon hypertrophic conversion by BMP4 (Hyp − T3 + BMP).

**Figure 6:**
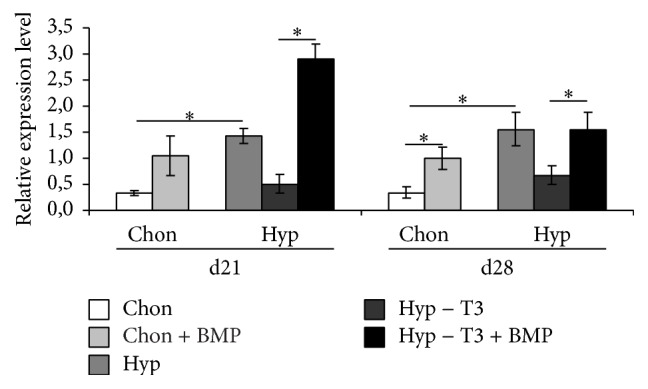
Gene expression analysis of BAMBI normalized to HPRT in MSC pellet cultures after BMP4 treatment under chondrogenic (chon) and hypertrophy (hyp) enhancing conditions analysed by real-time PCR. BAMBI expression is increased under chondrogenic and hypertrophic conditions after BMP4 treatment. *n* = 4 different donors, ^*∗*^ *p* < 0.05.

BMP4 treatment significantly increases BAMBI expression on day 28 under chondrogenic conditions (nonsignificant increase on day 21). Under hypertrophic conditions BAMBI expression is significantly increased in MSC pellets that were treated with BMP4 compared to hypertrophic medium without T3 (hyp − T3) (Figure 6). In comparison to hypertrophic medium with T3 (hyp), we only could detect nonsignificantly increased gene expression levels upon BMP4 treatment (hyp − T3 + BMP) on day 21 but not day 28 (Figure 6). Both conditions, hyp and hyp − T3 + BMP, showed higher levels of BAMBI gene expression compared to BMP4 treatment under chondrogenic conditions.

### 3.5. Gene Expression of BAMBI Is Downregulated by BMP Inhibition

According to these findings we further investigated whether these BAMBI expression patterns can be avoided by addition of the BMP inhibitor Noggin to hypertrophic (hyp) conditions. In order to estimate whether this inhibition was dose dependent we used two different doses of Noggin (10 and 100 ng/mL). For controls we added the same doses to chondrogenic controls.

**Figure 7:**
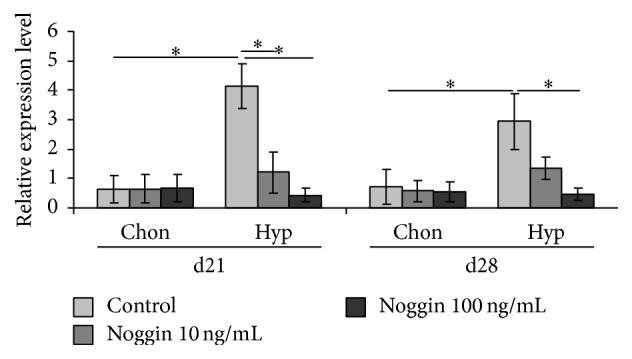
Gene expression analysis of BAMBI normalized to HPRT in MSC pellet cultures after Noggin treatment under chondrogenic (chon) and hypertrophy (hyp) enhancing conditions analysed by real-time PCR. Noggin treatment significantly decreases BAMBI expression under hypertrophic conditions. *n* = 4 different donors, ^*∗*^ *p* < 0.05.

Treatment with the BMP inhibitor Noggin does not alter BAMBI expression under chondrogenic conditions on day 21 and day 28. Under hypertrophic conditions, addition of 100 ng/mL Noggin significantly decreases BAMBI expression on day 21 and day 28 compared to hyp-conditions (Figure 7). However, decrease of BAMBI expression upon addition of 10 ng/mL Noggin is only significant on day 21, when compared to hyp-conditions. Besides the fact that BAMBI expression is downregulated under hypertrophic conditions and concurrent addition of the BMP inhibitor Noggin we could also show that this downregulation is dose dependent.

## 4. Discussion

### 4.1. Induction of Hypertrophy

Human MSCs are able to differentiate into a chondrogenic lineage. However some hypertrophic markers are expressed nevertheless, whether in short term culture or long term culture [28]. According to these findings it has been shown that cultured chondrogenic MSCs follow the intrinsic pathway of growth plate chondrocytes [12] and therefore inevitably become hypertrophic. This hypertrophic phenotype of chondrogenic differentiated MSCs can be experimentally enhanced by changing medium conditions from chondrogenic to hypertrophy enhancing medium as described previously [12, 14]. This change in medium conditions includes withdrawal of TGF-*β* and dexamethasone and the addition of the thyroid hormone T3. Using this in vitro hypertrophy model for MSCs we could significantly increase the hypertrophic phenotype of chondrogenic differentiated human MSCs. The enhancement of hypertrophy was clearly shown by an increased cell size, stronger collagen type X staining, and stronger ALP staining in hypertrophic MSC pellets. Hypertrophic markers are not exclusively expressed under prohypertrophic conditions but also under standard chondrogenic conditions even though to a lower degree. There are some ALP positive cells in the center of chondrogenic MSC pellets but ALP activity is mainly restricted to the periphery of the pellet. It was described previously that fibroblast-like cells surround MSC pellets [7]. Based on this finding, we suggest that the ALP positive ring around the pellet consists mainly of fibroblast-like cells rather than hypertrophic chondrocytes. After hypertrophic induction, areas of cellular hypertrophy as well as dedifferentiated areas were detected.

### 4.2. Gene Expression of BAMBI Is Increased upon Hypertrophic Conversion

BAMBI is a transmembrane protein with structural similarity to TGF-*β* and BMP receptor type I but lacks the intracellular kinase domain [21]. Thus, BAMBI is able to inhibit both TGF-*β* and BMP signalling. BAMBI has been shown to be coexpressed with BMP4 during xenopus and mouse embryogenesis [21, 22]. Further on BMP4 induces BAMBI expression through an evolutionary preserved promoter BMP responsive enhancer 7 (Bre7) [29]. Additionally also TGF-*β* is able to enhance BAMBI expression through SMAD 3/4 signalling [30].

We detected a significant upregulation of BAMBI under hypertrophic conditions using T3 and withdrawal of dexamethasone and TGF-*β* for hypertrophic conversion (Figure 3). The upregulation was proven for gene expression as well as on protein level by Western blotting. The final function of this strong upregulation of BAMBI however is still unclear. Preferential inhibition of TGF-*β* signalling diminishes the antihypertrophic effect of TGF-*β*, resulting in further enhancement of the hypertrophic phenotype. On the other hand, predominant interaction with BMP receptors could ameliorate the BMP induced terminal differentiation [23] in our prohypertrophic culture conditions. BAMBI may act as a negative feedback loop to inhibit BMP signalling in response to increased BMP4 expression.

### 4.3. Immunohistochemical Staining of BAMBI Is Increased upon Hypertrophic Conversion

The same observation of upregulation of BAMBI expression was conducted using immunohistochemistry (IHC) with primary antibodies against the BAMBI pseudoreceptor. Particularly the membrane binding nature of the pseudoreceptor, which is also known as a transmembrane receptor, was shown in higher magnifications of IHC. The membrane binding character was described earlier by several working groups [21, 31].

### 4.4. Gene Expression of BAMBI Is Induced by BMP4

To further clarify the induction of BAMBI expression we conducted an experiment in which hypertrophic conversion by T3 (hyp) was compared to hypertrophic conversion by BMP4 (hyp − T3 + BMP4). As evidenced by PCR BAMBI was increased, upregulated upon stimulation by BMP4. The induction of BAMBI expression by T3 is most probably also mediated by BMP. It was shown in previous works that Noggin and dorsomorphin as BMP inhibitors are capable of inhibiting T3 induced hypertrophy [23]. Therefore the effects of T3 on BAMBI expression will be most likely achieved by enhanced BMP expression and as a consequence increased BAMBI expression.

### 4.5. Gene Expression of BAMBI Is Downregulated by BMP Inhibition

This hypothesis is further supported by the finding that BAMBI expression in T3 induced hypertrophy is dose-dependently downregulated upon addition of the BMP inhibitor Noggin. This crosstalk between T3 triggered cell differentiation and BMP4 was shown in human MSCs [23] and chicken embryos [32], while other working groups suggested BMP2 as main mediator of hypertrophic conversion in chick limb buds [33].

It is still under discussion which exact temporospatial distribution BAMBI has during limb development and whether its role is pivotal or not. It seems that BAMBI, which is a negative regulator of chondrogenesis, might be a target in order to stabilize the chondrogenic phenotype of chondrogenically differentiated MSCs.

## 5. Conclusion

These experiments showed that the expression of the pseudoreceptor BAMBI is strongly upregulated under hypertrophy enhancing conditions in human MSCs. T3, BMP4, and Noggin treatment are able to modulate BAMBI expression. The function of BAMBI is not clear and needs to be investigated by functional experiments with knockdown and overexpression of BAMBI in the future.

## Supplementary Material

Supplemental Figure 1: Gene expression analysis of collagen type II and X normalized to HPRT in MSC pellet cultures under chondrogenic (chon) and hypertrophy enhancing (hyp) conditions analysed by real time PCR. Collagen type II expression is increased under chondrogenic conditions while collagen type X expression is increased under hypertrophic conditions at day 28.

## Figures and Tables

**Table 1:** 

Gene	Sequence (forward)	Sequence (reverse)	Concentration
HPRT	CGAGATGTGATGAAGGAGATGG	GCAGGTCAGCAAAGAATTTATAGC	150 nM
BAMBI	CGATGTTCTCTCTCCTCCCAG	AATCAGCCCTCCAGCAATGG	150 nM
