# Supplementary figures and images for: Expression of BMP and Actin Membrane Bound Inhibitor Is Increased during Terminal Differentiation of MSCs
Source: Stem Cells Int. 2016 Oct 23;2016:2685147. doi: 10.1155/2016/2685147 (PMC5097819; doi:10.1155/2016/2685147)

## Slide 1
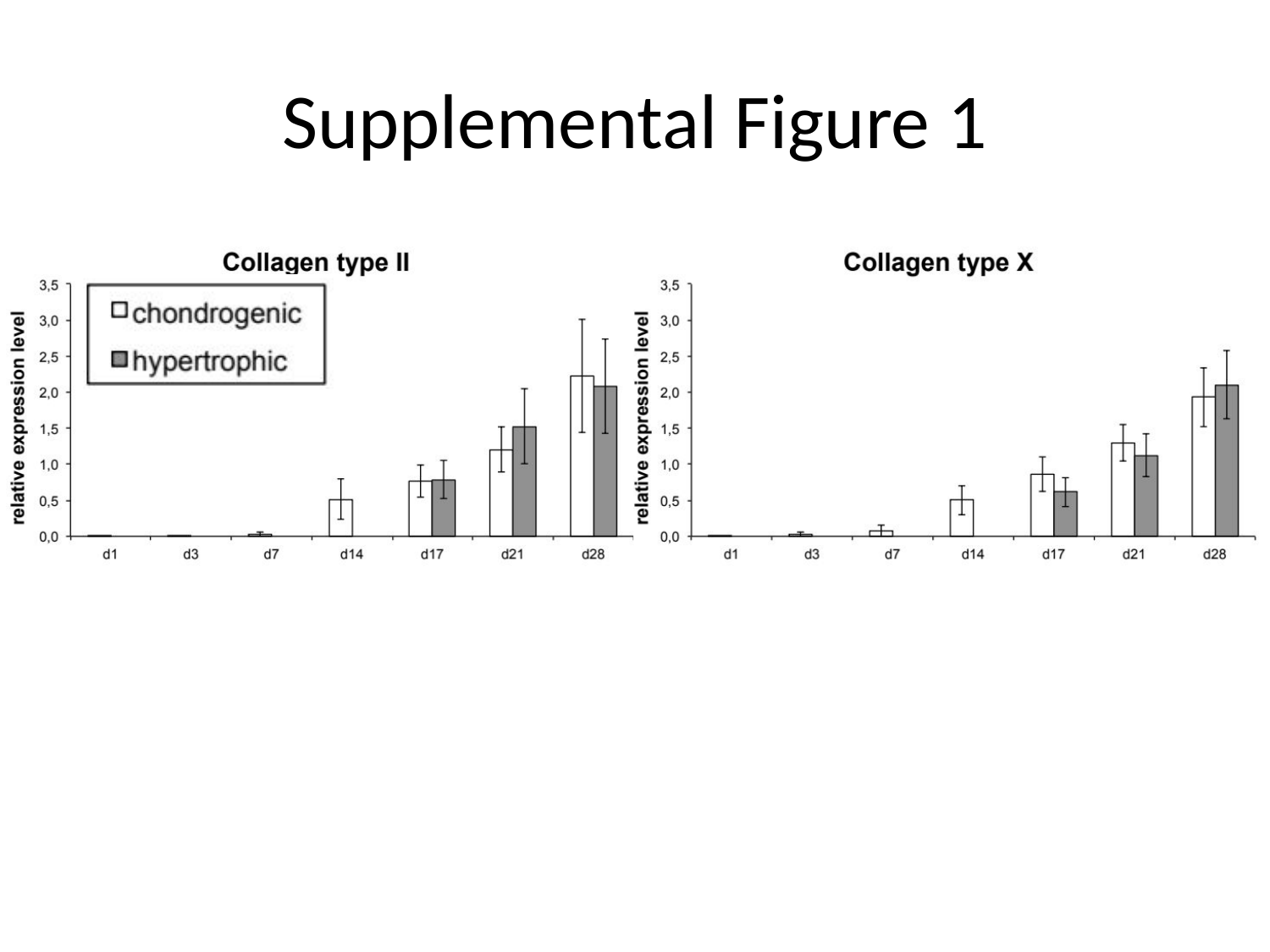

# Supplemental Figure 1

Supplement: Supplementary file 1 — Supplemental Figure 1: Gene expression analysis of collagen type II and X normalized to HPRT in MSC pellet cultures under chondrogenic (chon) and hypertrophy enhancing (hyp) conditions analysed by real time PCR. Collagen type II expression is increased under chondrogenic conditions while collagen type X expression is increased under hypertrophic conditions at day 28. [file 2685147.f1.pptx]
